# Carbolic Acid in the Treatment of Boils, Whitlows and Abscesses

**Published:** 1868-12

**Authors:** C. J. Cleborne

**Affiliations:** Surgeon U. S. N.


### Carbolic Acid in the Treatment of Boils, Whitlows and Abscesses.

BY C. J. CLEBORNE, M. D., SURGEON U. S. N.

As carbolic acid is exciting so much attention at the present time, I will give my experience with that article in the treatment of whitlows, boils and abscesses. During the past year I have had an unusually large number of these cases on board ship, and being dissatisfied with the usual mode of treatment, I determined to try the effect of carbolic acid. This I did by making a free opening as soon as fluctuation could be detected, and when all of the pus had been discharged by gentle pressure, I either injected or swabbed out the cavity with the ordinary liquid carbolic acid of the shops, after which I applied a cold water dressing. By this treatment further suppuration was prevented, and the wound healed so rapidly that the patient returned to duty in two or three days.

In some cases, after evacuating the pus, and using the acid, I drew the edges of the wound together with isinglass plaster, and in twenty-four hours it entirely healed. My experience, so far, has been with ordinary *undiluted* liquid carbolic acid (not carboline,) and the results have been so satisfactory that it deserves a trial in similar cases. In the treatment of gonorrhœa, I have not been satisfied with the liquid carbolic acid. As an injection it caused too much pain, and seemed to aggravate the symptoms when used even in the proportion of two to five drops to the ounce of water. These objections, it is said, do not apply to the crystalized acid of Merck, or the chemically pure article of Calvert, which may be used for this purpose in the proportion of two to five grains to the ounce of oil of almonds, or diluted glycerine.

